# Mucosal Immunogenicity of Genetically Modified *Lactobacillus acidophilus* Expressing an HIV-1 Epitope within the Surface Layer Protein

**DOI:** 10.1371/journal.pone.0141713

**Published:** 2015-10-28

**Authors:** Akinobu Kajikawa, Lin Zhang, Alora LaVoy, Sara Bumgardner, Todd R. Klaenhammer, Gregg A. Dean

**Affiliations:** 1 Department of Applied Biology and Chemistry, Tokyo University of Agriculture, Tokyo, Japan; 2 Department of Microbiology, Immunology, and Pathology, College of Veterinary Medicine and Biomedical Sciences, Colorado State University, Fort Collins, Colorado, United States of America; 3 Center for Comparative Medicine and Translational Research, College of Veterinary Medicine, North Carolina State University, Raleigh, North Carolina, United States of America; 4 Department of Food, Bioprocessing, & Nutrition Sciences, North Carolina State University, Raleigh, North Carolina, United States of America; Instituto Butantan, BRAZIL

## Abstract

Surface layer proteins of probiotic lactobacilli are theoretically efficient epitope-displaying scaffolds for oral vaccine delivery due to their high expression levels and surface localization. In this study, we constructed genetically modified *Lactobacillus acidophilus* strains expressing the membrane proximal external region (MPER) from human immunodeficiency virus type 1 (HIV-1) within the context of the major S-layer protein, SlpA. Intragastric immunization of mice with the recombinants induced MPER-specific and S-layer protein-specific antibodies in serum and mucosal secretions. Moreover, analysis of systemic SlpA-specific cytokines revealed that the responses appeared to be Th1 and Th17 dominant. These findings demonstrated the potential use of the *Lactobacillus* S-layer protein for development of oral vaccines targeting specific peptides.

## Introduction

HIV-1 is predominantly transmitted at mucosal surfaces, but vaccine design and evaluation have focused primarily on systemic immune responses. The mucosal immune system is, in many respects, independent of the systemic immune system. In humans, 90% of intestinal and 50% of vaginal IgA is produced locally and induction of mucosal immunity is best achieved via mucosal infection or vaccination [1–3]. Passive transfer studies using broadly neutralizing antibodies (BnAb) have shown protection against mucosal transmission (reviewed in [4]). Induction of BnAb has proven extraordinarily difficult because neutralizing epitopes are often structurally complex and difficult to faithfully recapitulate, long-term immune maturation is needed to acquire the extensive hypermutation described for most neutralizing IgG, and some neutralizing antibodies show autoreactivity [5,6]. However, BnAb may not be essential for protection at the mucosa.

There is strong evidence that protection by IgA typically does not rely on classical virus neutralization. IgA can sequester virus in mucus, sterically hinder binding to mucosal epithelia, and target virus for destruction via the polymeric immunoglobulin receptor (pIgR). There is also growing interest in the protective potential of non-neutralizing IgG [7–10]. Mechanisms of non-neutralizing protection include antibody-dependent cellular cytotoxicity, antibody-dependent cell-mediated virus inhibition, and other innate immune functions such as phagocytosis that are mediated by the Fc domain of the antibody. Whether mucosal vaccination can induce a protective antibody response of any sort against HIV-1 is uncertain.

Lactobacilli are an important group of Gram positive lactic acid bacteria used for food preservation, food bioprocessing and as probiotics. Lactobacilli are increasingly under investigation as biologic vaccine vectors. Proof of principle studies have been performed using recombinant lactobacilli as oral vaccines against tetanus toxin, anthrax, rotavirus, *Brucella aborus*, SARS Coronavirus, human papilloma virus, *Helicobacter pylori* and others (reviewed in [11–13]). In 2003, Xin and colleagues employed recombinant *Lactococcus lactis* to induce HIV-specific immune responses [14]. While this report clearly demonstrated the potential of lactic acid bacteria as vaccine vectors against HIV-1, there were two concerns with the approach that was employed. First, cholera toxin was used as an adjuvant and is not acceptable for use in humans. Second, the HIV-1 IIIB Env V2-V4 loop was used as the immunogen and is unlikely to induce a broadly protective immune response. Thus, alternative adjuvants and antigen design and expression are needed for a successful anti-HIV vaccine using lactic acid bacteria.

We and others have shown that several cell surface components of the probiotic bacteria are recognized by immune cells via pattern recognition receptors [15]. In particular, lipoteichoic acid, peptidoglycan (PG), and muramyl dipeptide, the subcomponent of PG, are known as the major immune stimulators recognized by the heterodimeric Toll-like receptor (TLR) 2/6 and nucleotide-binding oligomerization domain 2 (NOD2), respectively [16–18]. This capacity to interact with the innate immune system explains why lactobacilli can effectively induce mucosal IgA (reviewed in [19]).

The probiotic strain *Lactobacillus acidophilus* NCFM is particularly promising as an oral vaccine vector because: (1) it is acid and bile tolerant; (2) it expresses mucus-binding proteins and associates with the intestinal mucosa; and (3) it binds to dendritic cells (DCs) through DC-specific intercellular adhesion molecule 3 (ICAM-3)-grabbing nonintegrin (DC-SIGN) and other pattern recognition receptors described above [20]. Proof of principle has been demonstrated by Mohamadzadeh et al., who constructed recombinant *L*. *acidophilus* producing the *Bacillus anthracis* protective antigen and succeeded in inducing protective immunity in a murine model [21].

For construction of recombinant *L*. *acidophilus* as a vaccine candidate, there are three strategies for the subcellular distribution of antigens: cytoplasmic accumulation, secretion, and cell surface display [12,22]. In this study, we inserted a linear epitope from the membrane proximal external region (MPER) of HIV-1 into the highly expressed bacterial surface layer protein (SlpA) of *L*. *acidophilus*, as a prototype oral mucosal vaccine platform, and assessed immunogenicity in a mouse model.

## Materials and Methods

### Ethics statement

This study was carried out in strict accordance with the recommendations in the Guide for the Care and Use of Laboratory Animals of the National Institutes of Health, the US Public Health Service Policy on Humane Care and Use of Laboratory Animals, and the Association for Assessment and Accreditation of Laboratory Animal Care International (AAALAC). Protocol #11-3041A was approved by the Colorado State University Institutional Animal Care and Use Committee which operates under a currently approved Assurance #A3572-01. Animal welfare and health was monitored daily and in instances where medical intervention was not effective, animals were humanely euthanized and every effort was made to minimize suffering.

### Bacterial strains and culture conditions

*Lactobacillus acidophilus* NCK1909 and derivative strains were grown statically in MRS broth (BD Diagnostics, Sparks, MD) alone or supplemented with 2 or 5 μg/ml of erythromycin (Em) and 5 μg/ml of chloramphenicol (Cm) at 37°C. MRS (1.5% agar) plates with or without antibiotics were incubated anaerobically. *Escherichia coli* EC101 and other strains were grown aerobically with shaking in LB medium (BD Diagnostics) with or without 200 μg/ml of Em and 40 μg/ml of kanamycin (Km) at 37°C. The bacterial strains used in this study are listed in S1 Table.

### DNA manipulation and recombination

A modified *slpA* gene including in-frame MPER peptide-encoding sequence and flanking regions was generated by overlap PCR. Approximately 1 kb DNA fragments of the upstream and downstream regions were amplified using primer pairs AK_63 and AK_55, or AK_54 and AK_64. Chromosomal DNA of *L*. *acidophilus* NCFM was used for template DNA. The PCR products were applied to the second round of PCR along with AK_63 and AK_64. The connected 2 kb fragment was treated with *Bam*HI and *Hin*dIII followed by ligation with the digested pTRK935. The resulting plasmid, pTRK1053, was introduced into *L*. *acidophilus* NCK1910 by electroporation. The gene replacement by double crossing-over was performed as previously described [23]. The mutation in *slpA* was validated by PCR. A DNA fragment encoding matured mouse IL-1β was fused with the signal sequence of *L*. *acidophilus mub* gene (locus_tag: LBA1709) by overlap PCR. Each DNA fragment was amplified using either PCK_1 & AK_80 with *L*. *acidophilus* chromosomal DNA or AK_79 & PCK_2 with total mouse cDNA. The fusion DNA was inserted between the *Eco*RI and *Eag*I sites of pTRK882 [24]. The established plasmid, pGAD17, was introduced into *L*. *acidophilus* NCK2208, the strain displaying MPER. The nucleotide sequences of the primers are described in S2 Table.

### SDS-PAGE and Western blot

Total proteins of bacteria were prepared by bead beating in a lysis buffer (8M urea in 50 mM Tris-Cl, pH 8.0). In some experiments, culture supernatant was collected and then concentrated by adding 1/10 volume of trichloroacetic acid. Purified S-layer proteins were prepared as previously described. [23] The proteins were separated in NuPAGE 4–12% Bis-Tris Gel with MOPS-SDS running buffer (Life Technologies, Grand Island, NY). Gels were either stained with Coomassie brilliant blue (CBB) or applied to electroblot against Immobilon-P membrane (EMD Millipore, Billerica, MA). The protein blot was incubated with 1% bovine serum albumin (BSA) in phosphate buffered saline (PBS) including anti-HIV-1 gp41 monoclonal IgG, 2F5 [25]. As the secondary antibody, horseradish peroxidase (HRP)-conjugated anti-human IgG (Cell Signaling Technology Inc, Danvers, MA) was used. Signal was detected using ECL Plus (GE Healthcare Life Sciences, Piscataway, NJ) and visualized by Molecular Imager VersaDoc (Bio-Rad Laboratories, Hercules, CA).

### Verification of MPER expression

Bacterial cells from overnight cultures were treated with 2F5 antibody solution (10 μg/ml in PBS supplemented with 1% BSA) and then incubated with Alexa 488-conjugated anti-human IgG (Life Technologies) solution (4 μg/ml). Forward scatter (FSC), side scatter (SSC), and fluorescence intensity of stained cells suspended in PBS were analyzed with LSR II flow cytometer and FACSDiva software (BD Biosciences, San Jose, CA).

### Immunization and sampling of mice

Female Balb/c mice (8–10 weeks old) were immunized intragastrically (i.g.) with genetically modified *L*. *acidophilus* strains. Bacterial cells were freshly prepared from overnight culture and suspended in i.g. buffer containing NaHCO_3_ and soybean trypsin inhibitor [26]. Three daily doses of the bacterial suspensions (2×10^9^ CFU in 200 μl) were administered to mice (6 mice/group) on weeks 0, 2, 4, and 6. In another experiment, mice (12 mice for a group receiving GAD19, a MPER & IL-1β-co-expressing strain, and 4 mice for other groups) received an additional 4 sets of doses at weeks 8, 10, 12, and 14. Mice were euthanized two weeks after the last immunization. Blood was collected from tail vein periodically or by cardiac puncture terminally. Cecal contents were suspended in PBS supplemented with 1% BSA and 0.05% Tween-20, then supernatants were collected. Vaginal lavage was prepared by suspending vaginal fluid with PBS using pipet. Insoluble debris was removed by centrifugation. These samples were stored at -80°C. Spleen, Peyer’s patches (PPs), colon, and female reproductive tract (FRT) were also collected. Mice were housed and cared for in accordance with Association for the Assessment of Laboratory Animal Care Standards and Institutional Animal Care and Use Committee guidelines of Colorado State University (protocol #11-3041A).

### Preparation of cell suspensions and B cell analysis

Single cell suspensions of spleen and PP cells were prepared by mashing tissues in GentleMACS dissociator (Miltenyi Biotec, Auburn, CA) and filtering through cell strainers. Isolation of lymphocytes from colon and FRT was as previously described [27] with the following modifications. Mucus and epithelium were removed in PBS supplemented with 1 mM dithiothreitol and 5 mM EDTA. The tissues were then cut into small pieces, suspended in digestion medium, and applied to GentleMACS dissociator. After 30 min incubation at 37°C, additional dissociation was applied and the cell suspensions were transferred to new tubes through cell strainers. Purity and viability of isolated immune cells were validated by flow cytometry and trypan blue staining. B cells were analyzed by staining with anti-mouse CD45 (FITC), CD38 (PECy7), and CD19 (Pacific Blue) (BioLegend, San Diego, CA).

### Enzyme-Linked ImmunoSorbent Assay (ELISA) and Enzyme-Linked ImmunoSpot (ELISpot) assay

Microplates (96-well) were coated overnight with 1 μg/ml of synthetic 17-mer MPER peptide, GNEQELLELDKWASLWN (Bio-Synthesis Inc, Lewisville, TX), SlpA, or the modified SlpA. After blocking with 1% BSA in PBS, serially diluted sera were added. HRP-conjugated anti-mouse IgG or IgA were used as the detection antibodies. Color development with 3,3’,5,5’-tetramethylbenzidine (TMB) was terminated with sulfuric acid and absorbance at 450 nm was measured. To determine endpoint titer, sera from naïve mice were included in the assay and the mean value plus 3 standard deviations (SD) was applied as the cut-off value. Mouse Ig Isotyping ELISA Ready-Set-Go! (eBioscience, San Diego, CA) was used to determine class and subclass of anti-MPER Abs. Synthetic biotinylated 17-mer MPER (Bio-Synthesis Inc) was used for antigen-specific recognition. IL-1β secreted by *L*. *acidophilus* GAD19 and IL-6 released from stimulated murine splenocytes and Peyer’s patch cells were quantified by ELISA (eBioscience). Antigen-specific IgA-producing cells in mucosal tissues were detected by ELISpot as previously described [28].

### Multiple cytokine quantification

This procedure was performed in accordance with a previously described method with minor modification [29,30]. To determine cytokine responses, 1 × 10^6^ splenocytes were stimulated in 96-well plates with 5 μg/ml of 17-mer MPER or 10 μg/ml of S-layer protein of *L*. *acidophilus* NCFM for 72 hours. Multiple cytokines in culture supernatants were quantified with a customized Milliplex Map kit (EMD Millipore). Briefly, antibody-immobilized beads were incubated with 1 to 5 diluted culture supernatants. The beads were subsequently treated with detection antibodies as well as PE-conjugated streptavidin and analyzed by use of a Bio-Plex system (Bio-Rad Laboratories).

### Statistical analysis

Student’s t-test and Steel-Dwass test using JMP 10 software (SAS Institute Inc., Cary, NC) was applied for unpaired or multiple comparisons. Significant differences were defined as *P*<0.05.

## Results

### Gene replacement in *L*. *acidophilus* NCK1909 with modified slpA

**Fig 1:**
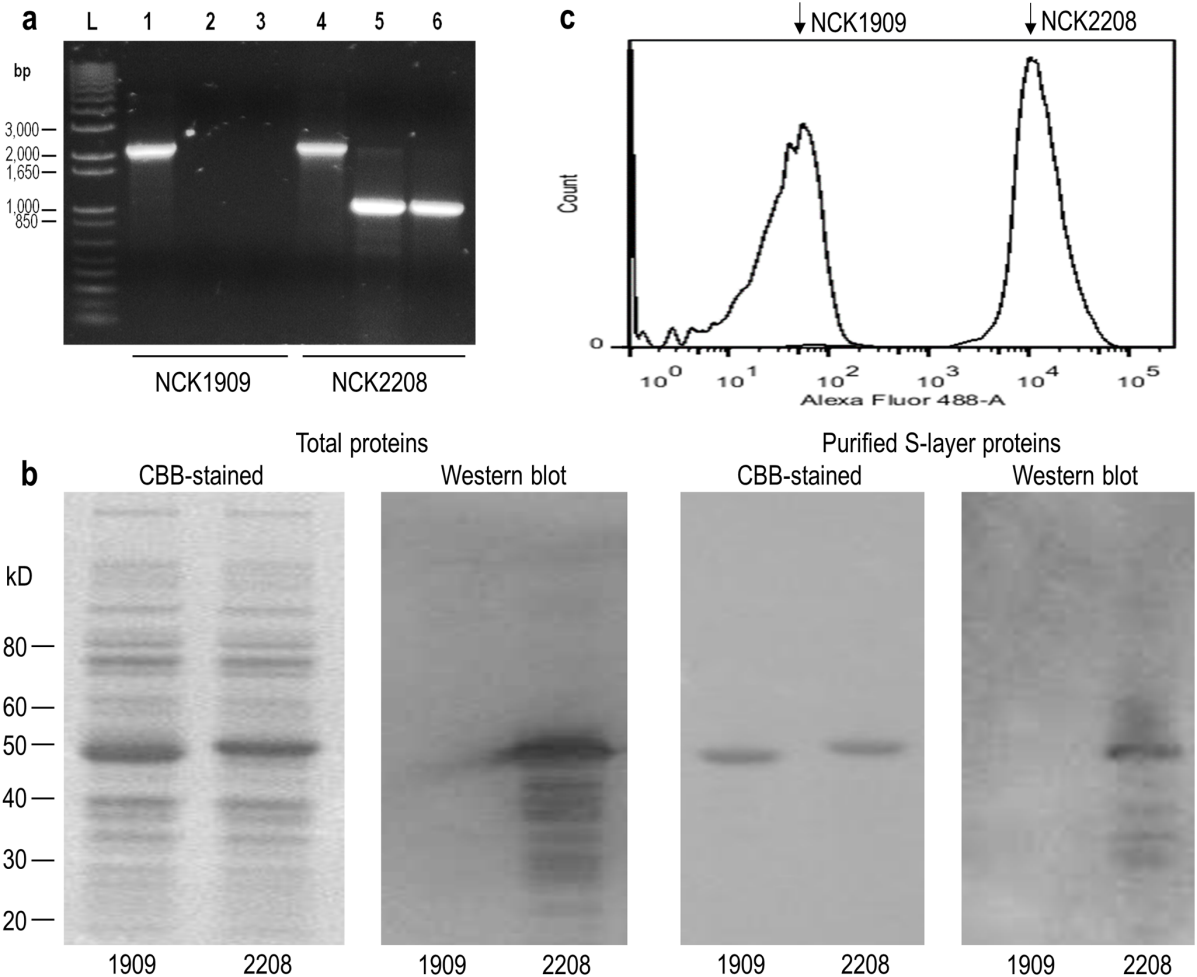
Validation of genetically modified *L*. *acidophilus* producing MPER-displaying S-layer proteins. The *L*. *acidophilus slpA* gene in NCK1909 was replaced with the modified *slpA* gene including MPER-encoding sequences by homologous recombination in NCK2208. (a) The gene replacement of *slpA* with the modified *slpA* was confirmed by PCR. L, DNA ladder marker. Amplified DNA fragments using primers, AK_62 and AK_65 (lane 1 and 4), AK_62 and AK_57 (lane 2 and 5), or AK_56 and AK_65 (lane 3 and 6). (b) Detection of the MPER epitope in S-layer (SlpA) protein using 2F5 mAb. Total cell proteins and purified S-layer proteins of NCK1909 and NCK2208 were separated by SDS-PAGE. The gels were stained with CBB or blotted onto PVDF membrane followed by western blot analysis using 2F5 (anti-MPER monoclonal human IgG). (c) The exposed MPER epitope was detected by flow cytometry. The *L*. *acidophilus* strains labeled with 2F5 and Alexa Fluor 488-conjugated anti-human IgG were analyzed. Relative fluorescence intensity of each strain was shown as histogram plot.

A *slpA* modified strain derived from *L*. *acidophilus* NCK1909 was constructed by gene replacement. The resulting strain, *L*. *acidophilus* NCK2208, includes the 16-mer MPER-encoding sequence integrated into SlpA. To validate this mutation, chromosomal DNA was analyzed by PCR using primers that were either specific to sequences flanking the replaced region or specific to the inserted MPER-encoding sequence (S1 Fig). As shown in Fig 1a, similar sizes of DNA fragments were amplified in both wild type and mutant strains when the outer primers were applied. Meanwhile, MPER-specific primers amplified a specific band only from the mutant strain. These results confirmed the wild type *slpA* gene was replaced with the modified *slpA* gene in NCK2208.

### Production of modified SlpA and an additional heterologous protein

Total proteins and purified S-layer proteins prepared from NCK1909 and NCK2208 were separated by SDS-PAGE and stained with CBB. As shown in Fig 1b, the S-layer protein of NCK2208 exhibited a slightly greater molecular mass than that of NCK1909. Western blot analysis using mAb 2F5 specifically labeled the S-layer protein of NCK2208, but not NCK1909. Additional smaller bands are most likely degraded S-layer proteins. The bacterial cells were also analyzed by flow cytometry. NCK2208 exhibited strong fluorescence intensity (MFI 9,915) while NCK1909 showed only background fluorescence (MFI 15) (Fig 1c). These results indicate that the epitope recognized by mAb 2F5 was exposed on the cell surface of NCK2208. To provide an additional adjuvant effect, NCK2208 was transformed with a plasmid coding for the mature form of murine IL-1β in a secretory expression cassette, termed GAD19 (S2 Fig). To demonstrate biological activity of the recombinant cytokine, supernatants from GAD19 were added to mouse lymphocytes from Peyer’s patch and spleen and IL-6 levels were measured. As expected, IL-6 was induced by supernatants from the IL-1β-secreting strain, GAD19 (S2 Fig). Another derivative strain, GAD31, was constructed with pTRK882 as a reference strain (S1 Table).

### Adaptive immune responses elicited by intragastric immunization with the recombinant lactobacilli

**Fig 2:**
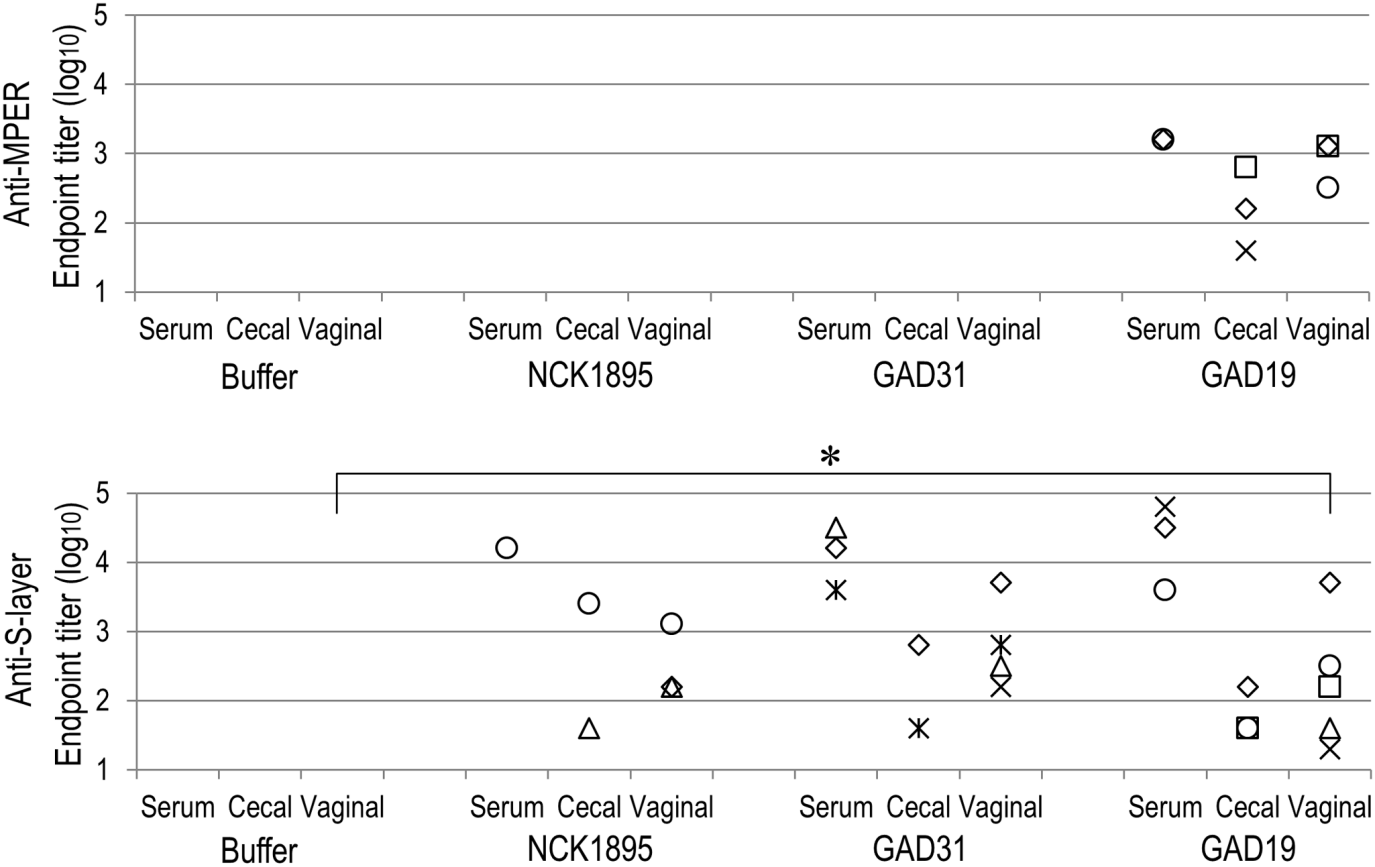
Induction of MPER- or S-layer protein-specific antibodies by oral immunization with *L*. *acidophilus* strains. The antigen specific serum IgG and mucosal IgA were titrated by ELISA. Each symbol represents an individual mouse. Values under detection limit (2 for IgG and 1 for IgA) are not shown in the chart. *P<0.05 (Steel-Dwass test).

**Fig 3:**
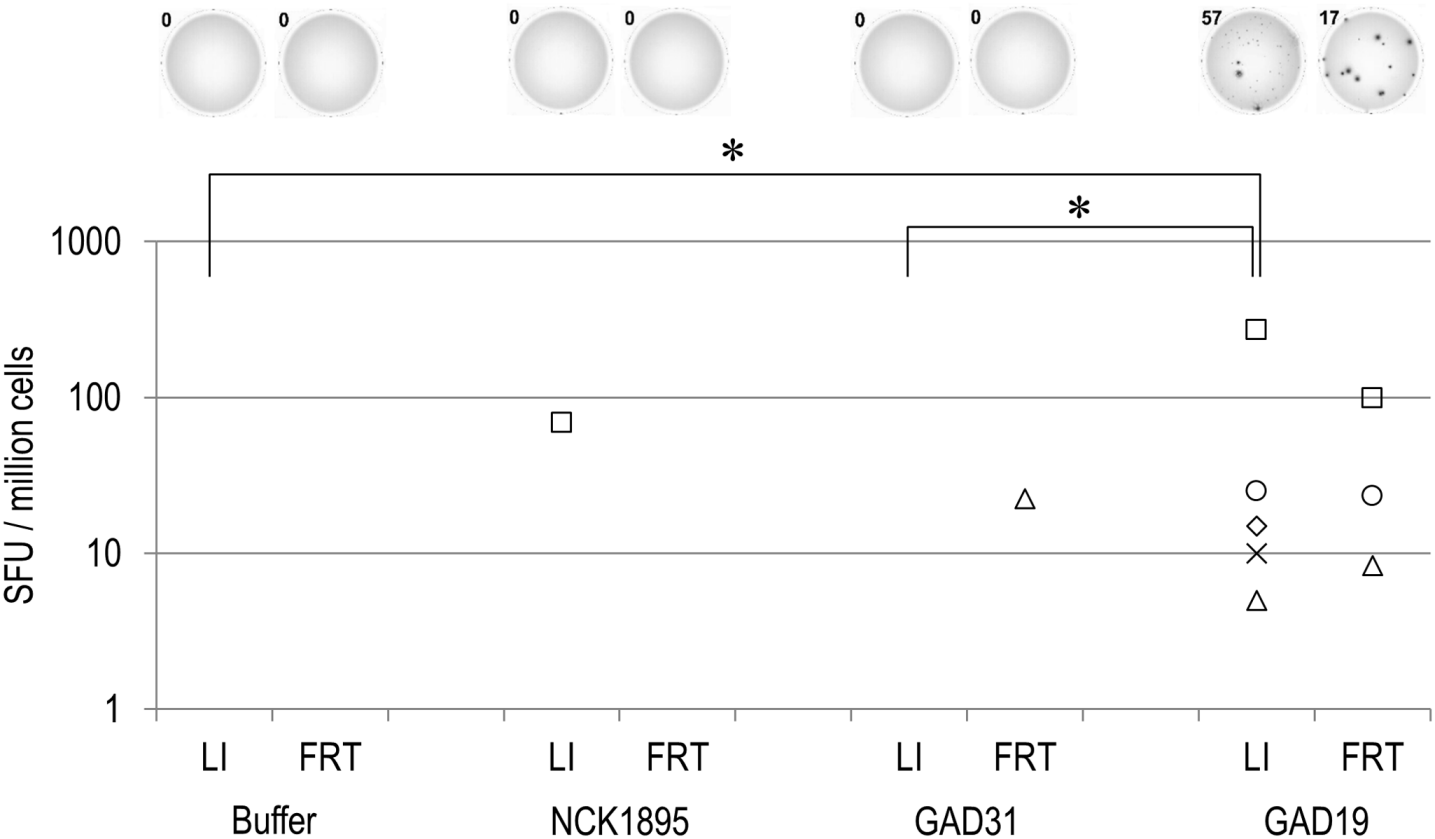
MPER-specific IgA producing cells in large intestine (LI) and female reproductive tract (FRT). Lymphocytes isolated from LI and FRT of immunized mice were analyzed by ELISpot assay. Representative images of the spots from each group are shown at the top. Each symbol represents an individual mouse. SFU, spot forming unit. *P<0.05 (Steel-Dwass test).

**Fig 4:**
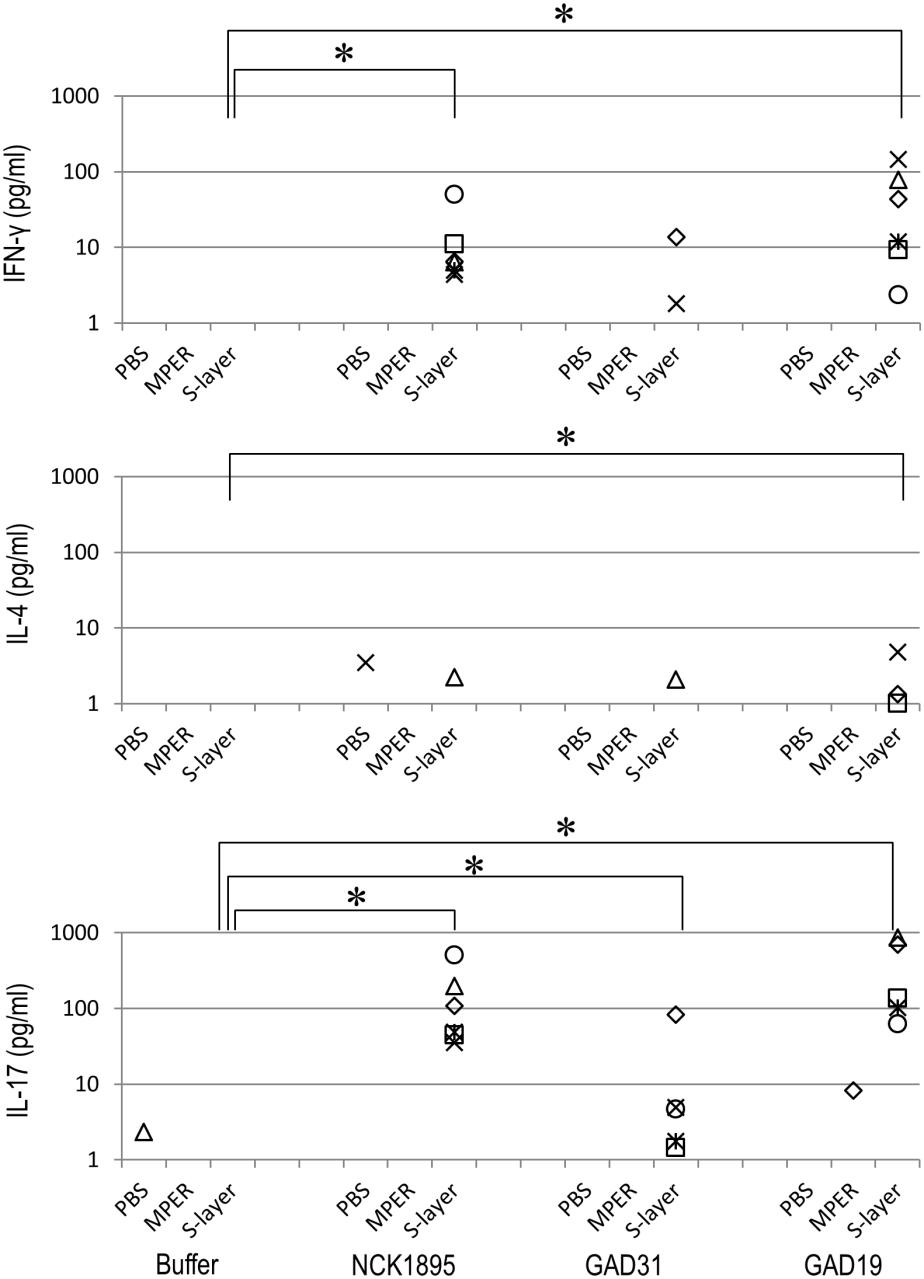
Detection of multiple cytokines produced by re-stimulated spleen cells. Culture supernatants of spleen cells isolated from immunized mice and incubated with MPER or SlpA for 72 hours. Each symbol represents an individual mouse. *P<0.05 (Steel-Dwass test).

The genetically modified strains of *L*. *acidophilus*, GAD19, GAD31, and NCK1895 were administered to mice via intragastric route. After the immunization, freshly isolated lymphocytes were analyzed to determine relative population of CD19^+^ CD38^+^ cells among CD45^+^ cells (S3 Fig). The relative B cell population were variable in GAD19-immunized group but there were no significant differences. As shown in Fig 2, MPER-specific antibody was detected only in mice receiving GAD19 (3/6). S-layer protein-specific antibodies were detected in all animals receiving lactobacilli. B cells producing MPER-specific IgA in large intestine and female reproductive tract were quantified by ELISpot assay. As shown in Fig 3, MPER-specific IgA-positive cells were detected almost exclusively in the GAD19-immunized group. These results suggest the adjuvant effect of IL-1β was required to enhance the immunogenicity of the MPER 16-mer contained in the SlpA. Cytokines produced by spleen cells in response to restimulation with MPER peptide or S-layer proteins were also analyzed (Fig 4). Cytokines were rarely detected in MPER-stimulated or non-stimulated splenocyte cultures. Meanwhile, moderate amounts of multiple cytokines, especially IL-17 and IFN-γ were released from spleen cells stimulated with S-layer protein in mice receiving the *L*. *acidophilus* strains.

### Induction of MPER-specific antibodies by long-term immunization

**Fig 5:**
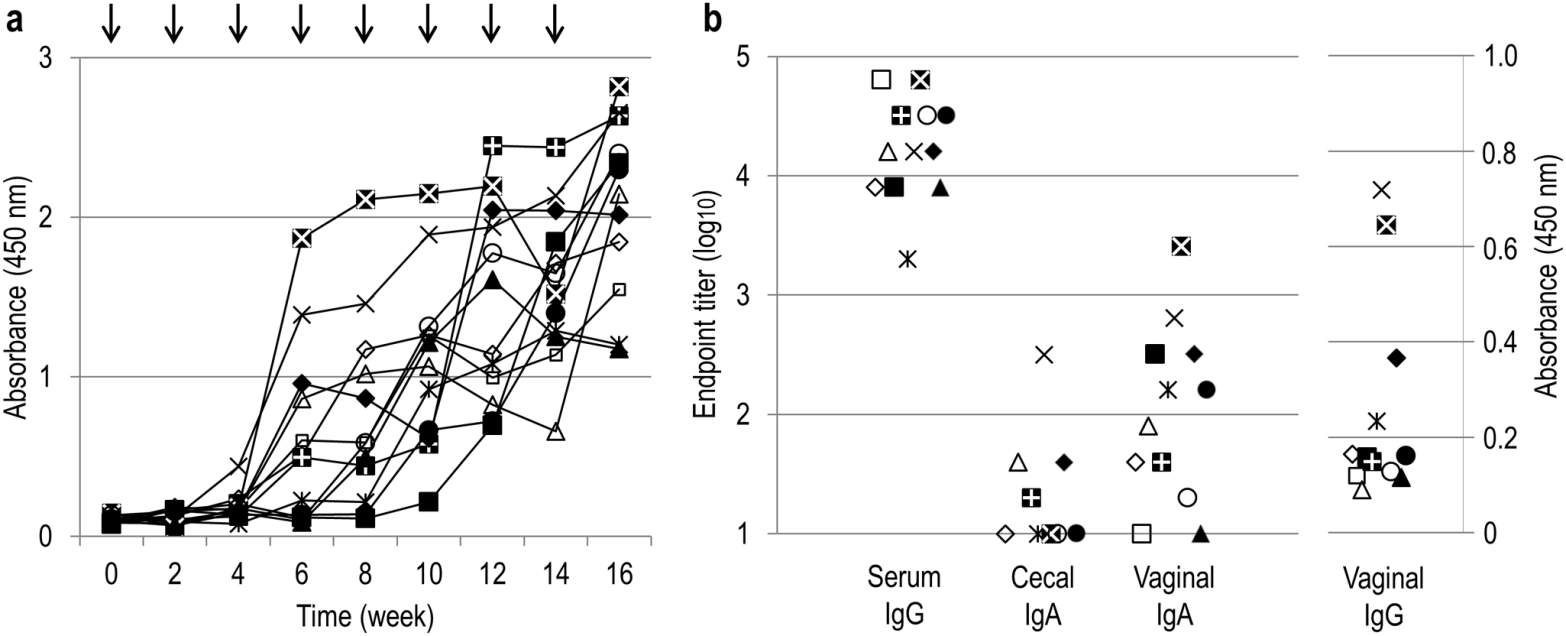
Induction of MPER-specific antibody production by long-term immunization. Mice received GAD19 orally every 2 weeks for 14 weeks. (a) Diluted serum (1/100) was analyzed by ELISA at each time point. Arrows represent timing of the gavage. (b) Endpoint titers (or absorbance at 450 nm) of MPER-specific serum IgG, cecal IgA, vaginal IgA, and vaginal IgG. Each symbol represents an individual mouse.

**Fig 6:**
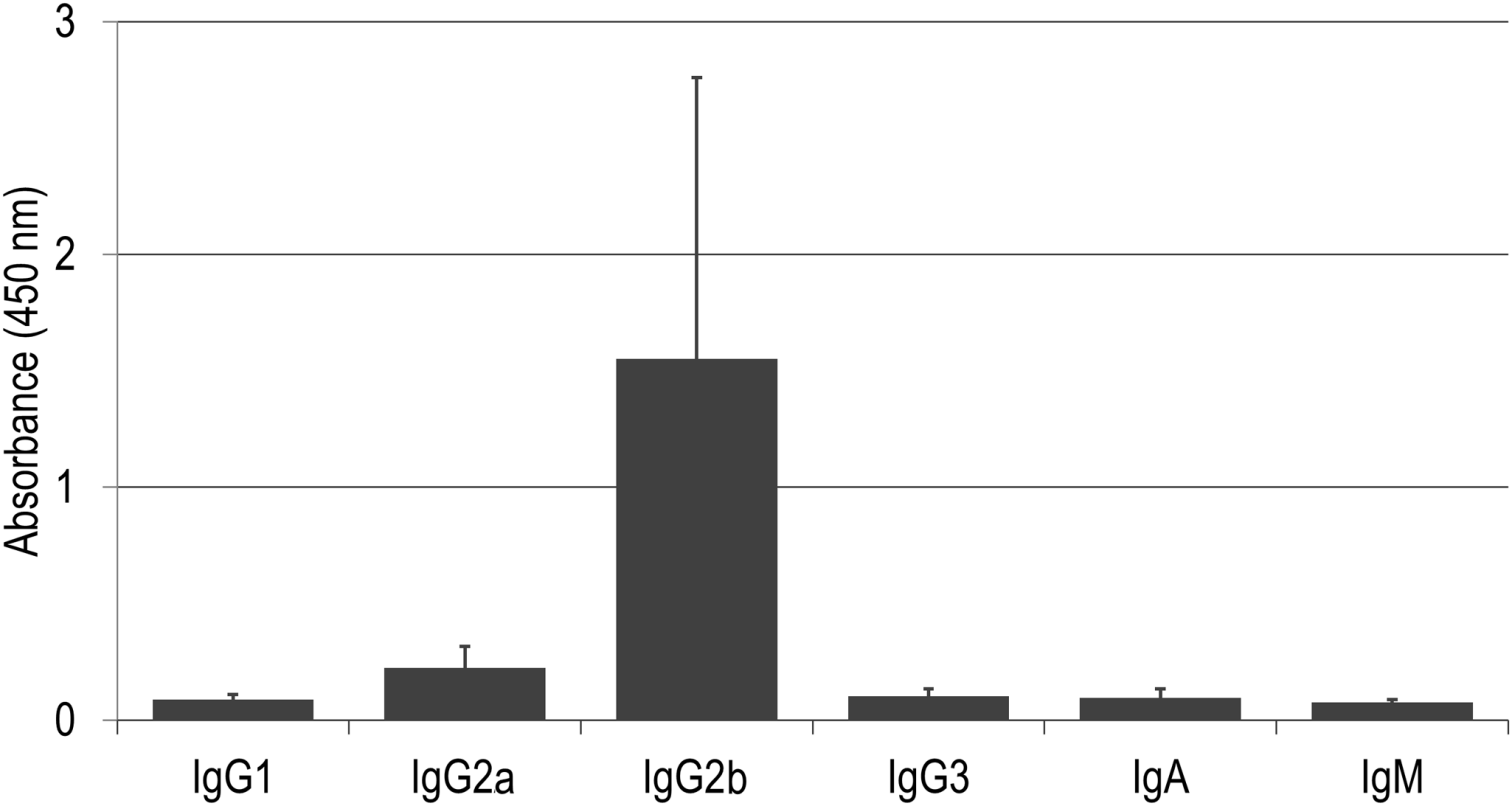
Typing of classes and subclasses of MPER-specific antibodies. Sera from GAD19-immunized mice were analyzed by ELISA. Each value plus SD (standard deviation) was shown.

Since the titers of antigen-specific Abs appeared not to have reached plateau at the terminal point (S4 Fig), a second study was performed with mice receiving a total of 8 immunizations. At week 16, all mice immunized with GAD19 developed MPER-specific IgG in sera and the response had not plateaued (Fig 5a). As shown in Fig 5b, endpoint titers of MPER-specific serum IgG were much higher than those at the first study. Mucosal IgA specific to MPER was also detected in most immunized mice. In some individuals, MPER-specific IgG was also present in vaginal lavage fluid. These results indicated that additional boosts with GAD19 evoked readily detectable levels of systemic and mucosal MPER-specific Ab responses. The additional boosts also showed that GAD31 was capable of inducing MPER-specific Ab production while no responses were shown in NCK1985 (S5 Fig). Isotype analysis of the MPER-specific serum antibody induced by GAD19 revealed that IgG2b was dominant, albeit only small part of anti-MPER could be detected due to low sensitivity of the assay (Fig 6).

## Discussion

S-layer proteins are dominating cell-surface components of some bacteria that serve as scaffolds for functional peptides. Because of their abundance, S-layer proteins may be a more efficient means to display specific vaccine epitopes as compared to other surface display approaches such as LPXTG-anchored proteins and flagella [14,31,32]. The present study shows that a mutant *L*. *acidophilus* displaying MPER was successfully established by modification of the *slpA* gene. The high frequency of the epitope on the bacterial surface was demonstrated by flow cytometry and immunoblot assay. Importantly, mAb 2F5 recognized the MPER peptides exposed on the S-layer proteins suggesting that the heterologous 16-mer peptide reproduced the corresponding structure of HIV-1. Presently, insertion of peptides longer than 19 amino acids into SlpA protein without destructive effects on the S-layer structure has been difficult ([33] and our unpublished data). Work is ongoing to successfully engineer the insertion of longer and/or multiple peptides. In previous studies, S-layer proteins of a different *L*. *acidophilus* strain and a *Lactobacillus brevis* strain were engineered to include a c-Myc epitope, although the immunological properties were not determined [33,34]. Scheppler et al. reported that immunization of mice with a recombinant *Lactobacillus johnsonii* strain expressing PrtB, the cell wall anchored proteinase of *Lactobacillus delbrueckii* subsp. *bulgaricus*, with an inserted mimotope of tetanus toxin induced antibodies specific to the bacterial cell and PrtB but not to the mimotope [35]. This emphasizes that display of epitopes on the bacterial surface does not guarantee immunogenicity. Hence, we investigated whether the MPER on SlpA could elicit specific immune responses *in vivo*.

In a preliminary experiment, *L*. *acidophilus* NCK2208 was only weakly immunogenic with no antibody response to MPER. To improve the mucosal immunogenicity of NCK2208, matured murine IL-1β was employed since IL-1 and IL-1 family proteins are known to act as mucosal adjuvants [36,37]. We previously showed that biologically active IL-1β can be produced and secreted by another recombinant *Lactobacillus* strain [38]. In the first round of i.g. immunization with the recombinant strain and reference strains, both MPER-specific Abs and the specific IgA-producing cells were detected exclusively in the group receiving the IL-1β-secreting strain. On the other hand, SlpA-specific responses did not rely on the cytokine. These results implied that the induction of MPER-specific but not SlpA-specific Abs was adjuvant-dependent. However, in the second trial where mice received four additional boosts, both *L*. *acidophilus* strains eventually elicited MPER-specific Ab responses regardless of IL-1β co-expression. This suggests that IL-1β was not essential for, but possibly expedited the specific immune responses. Additional studies are needed to confirm the adjuvant effect of IL-1β and better define the mechanism of action.

Although many studies have employed recombinant lactic acid bacteria for vaccine delivery, little information on anti-vector responses has been reported. The current study showed that repeated, high dose immunization with *L*. *acidophilus* evoked S-layer protein-specific antibodies and cytokine responses. Splenocytes isolated from mice immunized with the *L*. *acidophilus* strains were re-stimulated with purified S-layer proteins. Production of several cytokines was markedly upregulated, most notably, IFN-γ and IL-17. This suggests that the systemic immune responses specific to S-layer proteins were Th1 and Th17 dominant. Since the pattern of cytokine production in each group treated with *L*. *acidophilus* strains was similar regardless of SlpA-mutation or co-expression of IL-1β, those responses were likely attributed to the nature of the S-layer protein, per se. SlpA of *L*. *acidophilus* has previously been shown to induce cytokine production by dendritic cells via DC-SIGN in vitro [20]. Our current study reveals the role of the S-layer proteins in adaptive immune responses in vivo.

In contrast to S-layer proteins, in vitro restimulation of splenocytes with MPER peptide induced little or no cytokine production. This suggests the MPER peptide embedded in the S-layer protein did not stimulate a T cell response and that the MPER-specific antibody response was T cell independent. Isotype analysis revealed that the major subclass of MPER-specific antibody was IgG2b, which is known to be evoked in a T cell independent manner [39]. The involvement of TGF-β in IgG2b switching has previously been reported [40]. As mentioned above, S-layer proteins stimulate a Th17 response, which is known to require IL-6 and TGF-β. Taken together, TGF-β produced in response to S-layer proteins of *L*. *acidophilus* may drive or facilitate a T cell independent antibody response against MPER. This could be an important feature of the *L*. *acidophilus* vaccine platform given the growing general concerns that vector-induced T cell responses may enhance HIV-1 infection [41].

Prevention of HIV-1 transmission may be most achievable at the local mucosa where the natural bottleneck is greatest. The current study demonstrates that genetically engineered *L*. *acidophilus* can induce both mucosal and systemic antigen-specific antibodies by repeated mucosal immunization. However, the functional characteristics of the induced antibodies remain to be determined. Classical virus neutralization may not be essential if other mechanisms can reduce the likelihood of infectious virions contacting target cells. Several functional attributes of mucosal antibodies have been described for pathogen neutralization [42]. These include immune exclusion, intracellular neutralization, reverse-transcytosis, and immune targeting via the high-affinity IgA receptor (CD89) expressed on dendritic cells and neutrophils [43–45]. Furthermore, local elimination of early virus targets via antibody-dependent cellular cytotoxicity could create a one-two punch and provide a significant level of protection without the need for rapid immune activation. Clearly, it remains to be confirmed, in an appropriate animal model, whether recombinant *L*. *acidophilus* can induce a protective mucosal and systemic antibody response against HIV-1 without activating mucosal T cell targets.

## Supporting Information

S1 Fig The schematic map of wild/modified *slpA* gene and position of primers. The insertion site of MPER peptide in SlpA was chosen in accordance with the study of Smit et al. [46]. A 16-mer polypeptide of MPER (NEQELLELDKWASLWN), which was employed previously by Jain et al. [47], was selected for the insertion. The MPER peptide-encoding sequences were included in primers AK_54 and AK_55. A modified *slpA* gene (bottom) including MPER-encoding nucleotide sequences was generated from wild type *slpA* gene (top) using overlap PCR. Arrows with numbers represent primers. *P*, the promoter of *slpA* gene. T, the terminator of *slpA* gene. M, MPER-encoding nucleotides.
(TIF)

S2 Fig Secretion of matured murine IL-1β by GAD19. (a) Production of murine IL-1β was confirmed by western blot using anti-mouse IL-1β. Cell extracts of GAD19 and GAD31 (lane 1 and 3), culture supernatants (lane 2 and 4), and purified murine IL-1β (lane 5) are shown. (b) Biological activity of the recombinant IL-1β secreted by GAD19 was confirmed by induction of IL-6. Overnight cultures of recombinant lactobacilli were centrifuged and supernatants were sterilized by filtration. After quantification of IL-1β by ELISA, culture supernatants of GAD19 including 1 ng/ml of IL-1β (black bar) were added to Peyer’s patch or spleen cells of Balb/c mice and incubated for 72 hours. For references, the same volume of the culture supernatant of GAD31 (gray bar) and 1 ng/ml of purified IL-1β (open bar) were also tested. Values are means of duplicated assay and similar results were reproduced.
(TIF)

S3 Fig Relative population of CD38^+^CD19^+^ cells in mucosal tissues. Freshly isolated lymphocytes from LI (a) and FRT (b) tissues of immunized mice were labeled with anti-CD19, anti-CD38, and anti-CD45 Abs. CD45^+^ cells were gated and percentage of CD38^+^CD19^+^ cells were counted by FACS analysis. No significant difference was shown (P>0.05). LI: large intestine, FRT: female reproductive tract.
(TIF)

S4 Fig Time course of anti-MPER or anti-S-layer protein IgG responses in serum. Diluted sera (1/100 for MPER and 1/1000 for S-layer protein) were analyzed by ELISA at weeks 0, 2, 4, 6, and 8. Each symbol represents an individual mouse. Solid line, anti-MPER. Dotted line, anti-S-layer protein. Arrows indicate timing of the immunizations.
(TIF)

S5 Fig Induction of MPER-specific antibody production by long-term immunization. Mice were received the buffer, NCK1895, or GAD31 orally every 2 weeks. (a) Diluted serum (1/100) from each time point was analyzed by ELISA. Arrows represent timing of the gavage. Solid line, Buffer. Dotted line, NCK1895. Bold line, GAD31. (b) Endpoint titers of MPER-specific serum IgG, fecal IgA, and vaginal IgA. (c) Absorbance at 450 nm of MPER-specific vaginal IgG. Each symbol represents an individual mouse.
(TIF)

S1 Table Bacterial strains and plasmids. (DOCX)

S2 Table PCR primers. (DOCX)
