# Supplementary figures and images for: Mucosal Immunogenicity of Genetically Modified Lactobacillus acidophilus Expressing an HIV-1 Epitope within the Surface Layer Protein
Source: PLoS One. 2015 Oct 28;10(10):e0141713. doi: 10.1371/journal.pone.0141713 (PMC4624987; doi:10.1371/journal.pone.0141713)

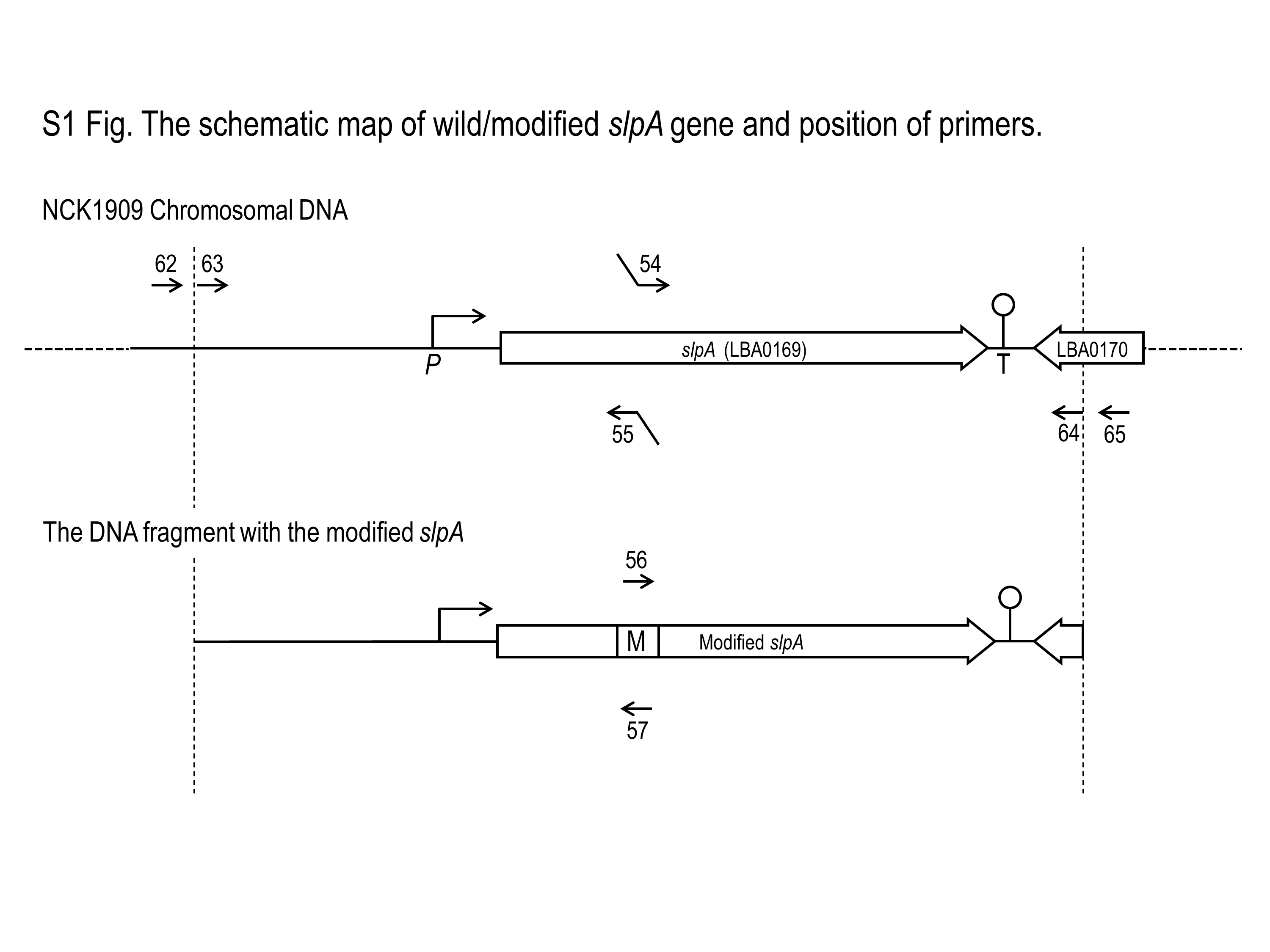

Supplement: S1 Fig — The insertion site of MPER peptide in SlpA was chosen in accordance with the study of Smit et al. [46]. A 16-mer polypeptide of MPER (NEQELLELDKWASLWN), which was employed previously by Jain et al. [47], was selected for the insertion. The MPER peptide-encoding sequences were included in primers AK_54 and AK_55. A modified slpA gene (bottom) including MPER-encoding nucleotide sequences was generated from wild type slpA gene (top) using overlap PCR. Arrows with numbers represent primers. P, the promoter of slpA gene. T, the terminator of slpA gene. M, MPER-encoding nucleotides. (TIF) [file pone.0141713.s001.tif]

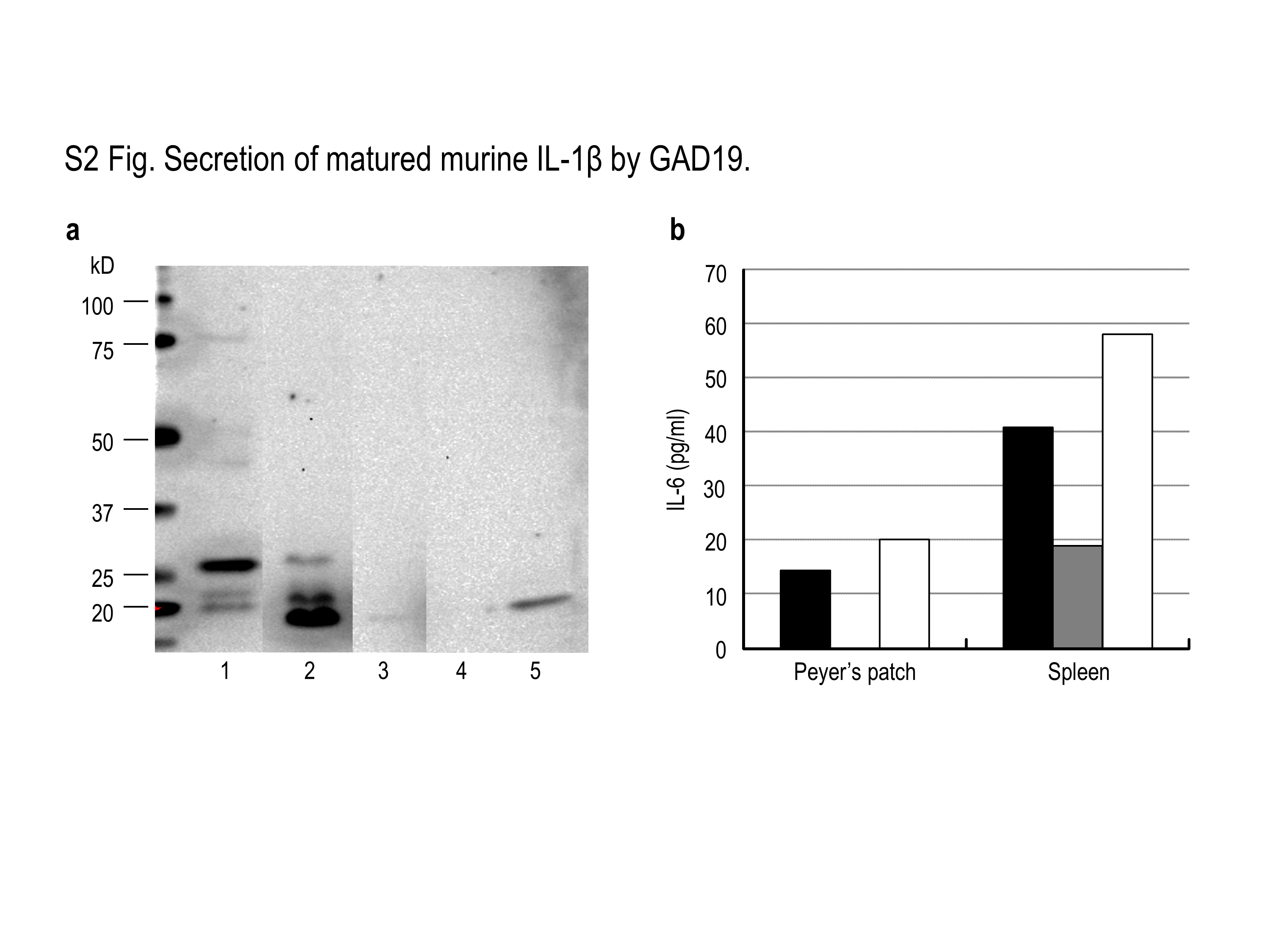

Supplement: S2 Fig — (a) Production of murine IL-1β was confirmed by western blot using anti-mouse IL-1β. Cell extracts of GAD19 and GAD31 (lane 1 and 3), culture supernatants (lane 2 and 4), and purified murine IL-1β (lane 5) are shown. (b) Biological activity of the recombinant IL-1β secreted by GAD19 was confirmed by induction of IL-6. Overnight cultures of recombinant lactobacilli were centrifuged and supernatants were sterilized by filtration. After quantification of IL-1β by ELISA, culture supernatants of GAD19 including 1 ng/ml of IL-1β (black bar) were added to Peyer’s patch or spleen cells of Balb/c mice and incubated for 72 hours. For references, the same volume of the culture supernatant of GAD31 (gray bar) and 1 ng/ml of purified IL-1β (open bar) were also tested. Values are means of duplicated assay and similar results were reproduced. (TIF) [file pone.0141713.s002.tif]

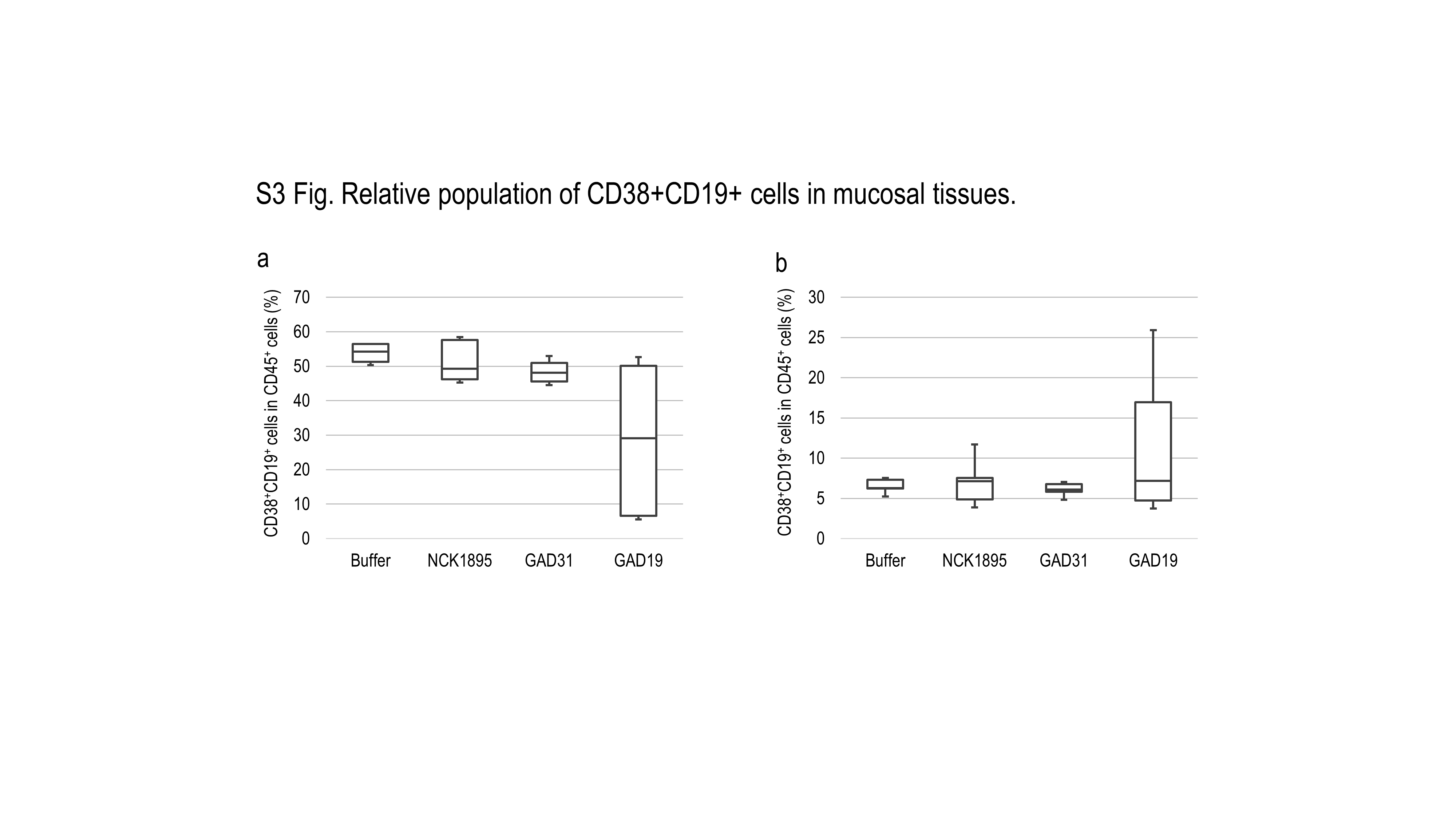

Supplement: S3 Fig — Freshly isolated lymphocytes from LI (a) and FRT (b) tissues of immunized mice were labeled with anti-CD19, anti-CD38, and anti-CD45 Abs. CD45+ cells were gated and percentage of CD38+CD19+ cells were counted by FACS analysis. No significant difference was shown (P>0.05). LI: large intestine, FRT: female reproductive tract. (TIF) [file pone.0141713.s003.tif]

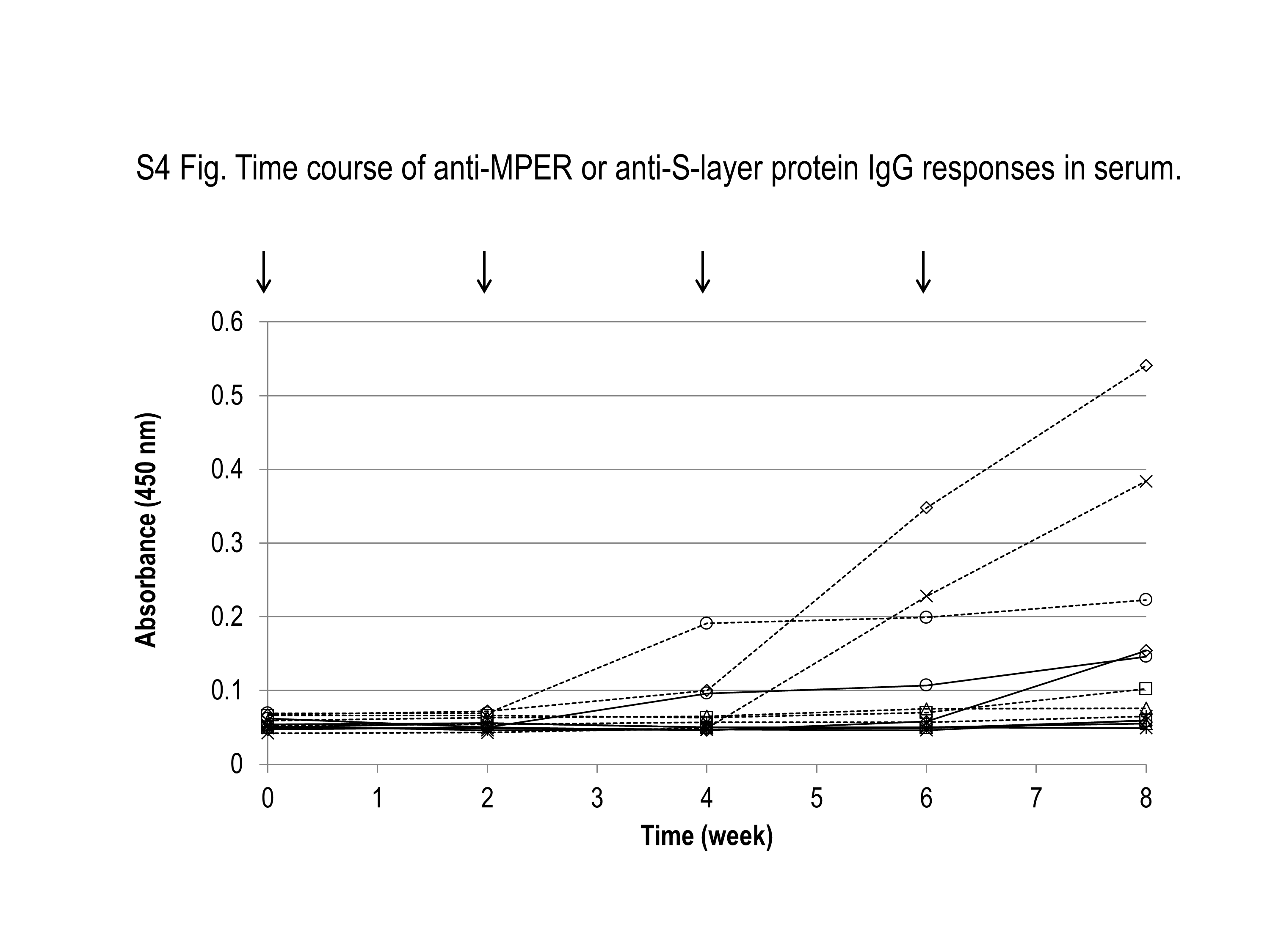

Supplement: S4 Fig — Diluted sera (1/100 for MPER and 1/1000 for S-layer protein) were analyzed by ELISA at weeks 0, 2, 4, 6, and 8. Each symbol represents an individual mouse. Solid line, anti-MPER. Dotted line, anti-S-layer protein. Arrows indicate timing of the immunizations. (TIF) [file pone.0141713.s004.tif]

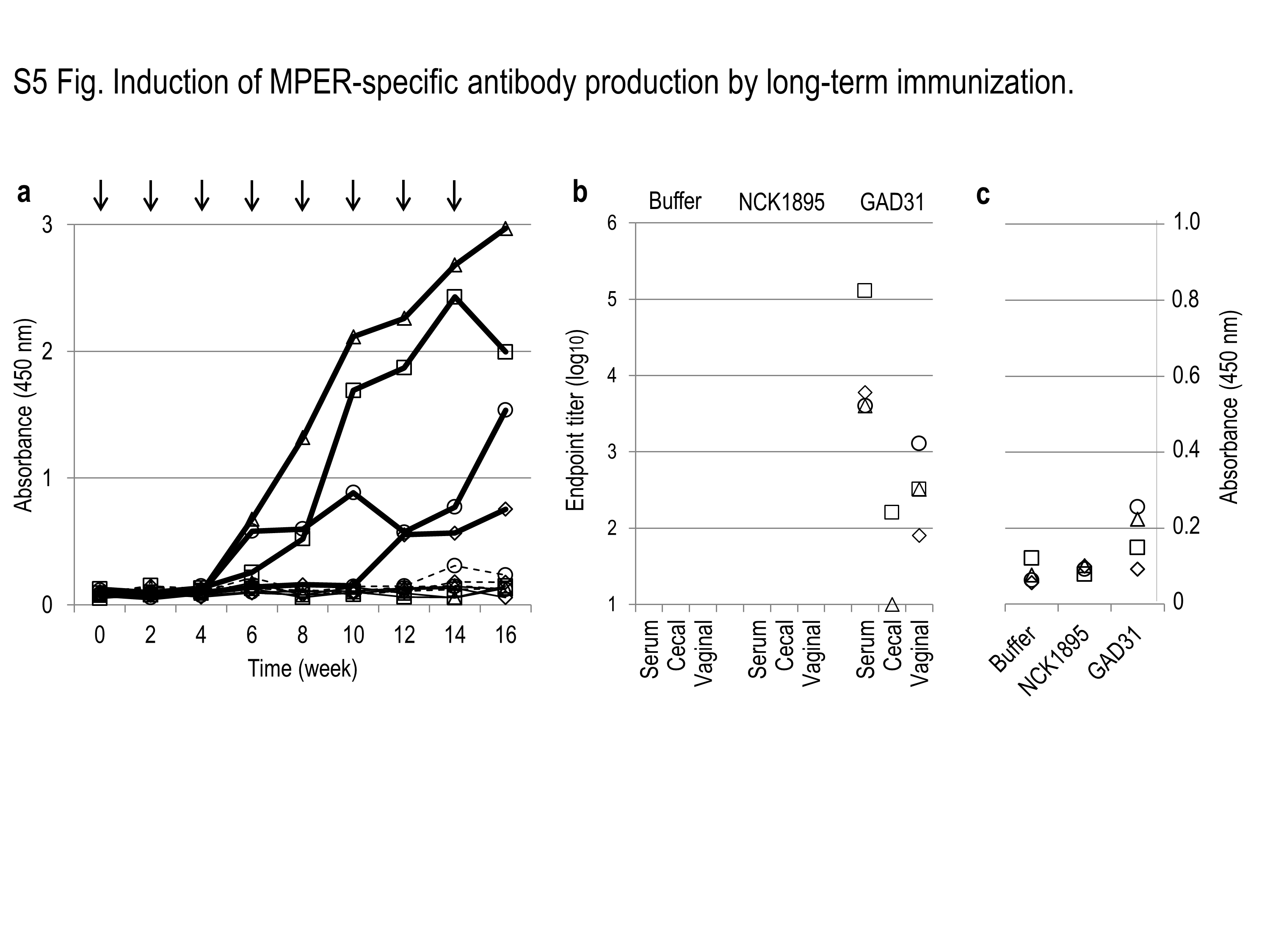

Supplement: S5 Fig — Mice were received the buffer, NCK1895, or GAD31 orally every 2 weeks. (a) Diluted serum (1/100) from each time point was analyzed by ELISA. Arrows represent timing of the gavage. Solid line, Buffer. Dotted line, NCK1895. Bold line, GAD31. (b) Endpoint titers of MPER-specific serum IgG, fecal IgA, and vaginal IgA. (c) Absorbance at 450 nm of MPER-specific vaginal IgG. Each symbol represents an individual mouse. (TIF) [file pone.0141713.s005.tif]
