# Supplementary material for: Mucosal Immunogenicity of Genetically Modified Lactobacillus acidophilus Expressing an HIV-1 Epitope within the Surface Layer Protein
Source: PLoS One. 2015 Oct 28;10(10):e0141713. doi: 10.1371/journal.pone.0141713 (PMC4624987; doi:10.1371/journal.pone.0141713)
Supplement: S1 Table — (DOCX) [file pone.0141713.s006.docx]

**S1 Table.** **Bacterial strains and plasmids.**

| Bacterial strains | Plasmids | Descriptions of bacterial strains and/or plasmids | References |
| --- | --- | --- | --- |
| *E. coli* EC101 |  | Cloning host for pTRK935 and derivatives, RepA^+^, Km^r^ | [1] |
| mc1061 |  | Cloning host for pTRK882 and derivatives | [2] |
| NCK2207 | pTRK1053 | EC101 derivative harboring pTRK935 with modified *slpA* inserted with MPER-encoding sequences, Km^r^, Em^r^ | [3], This study |
| GAD17 | pGAD17 | mc1061 derivative harboring pTRK882, an expression vector with *pgm* promoter, inserted with matured mouse IL-1β gene fused with signal sequence of *mub*, Em^r^ | [4], This study |
|  |  |  |  |
| *L. acidophilus* NCK1909 |  | NCFM derivative with *upp* gene deleted, producing wild type *slpA*, a reference strain for NCK2208 | [3] |
| NCK1910 | pTRK669 | NCK1909 derivative, a host for counterselective gene replacement, Cm^r^ | [3] |
| NCK2208 |  | NCK1909 derivative, modified *slpA*, producing SlpA-MPER | This study |
| NCK1895 | pTRK882 | NCFM derivative with the empty plasmid, producing wild type SlpA, a reference strain for GAD19 and GAD31, Em^r^ | [4] |
| GAD19 | pGAD17 | NCK2208 derivative producing SlpA-MPER, secreting mouse IL-1β , Em^r^ | This study |
| GAD31 | pTRK882 | NCK2208 derivative producing SlpA-MPER, Em^r^ | This study |

**References**

1. Law J, Buist G, Haandrikman A, Kok J, Venema G, Law J, et al. A system to generate chromosomal mutations in Lactococcus lactis which allows fast analysis of targeted genes . A System To Generate Chromosomal Mutations in Lactococcus lactis Which Allows Fast Analysis of Targeted Genes. Microbiology. 1995;

2. Casadaban MJ, Cohen SN. Analysis of gene control signals by DNA fusion and cloning in Escherichia coli. J Mol Biol. 1980;138: 179–207. doi:10.1016/0022-2836(80)90283-1

3. Goh YJ, Azcárate-Peril MA, O’Flaherty S, Durmaz E, Valence F, Jardin J, et al. Development and application of a upp-based counterselective gene replacement system for the study of the S-layer protein SlpX of Lactobacillus acidophilus NCFM. Appl Environ Microbiol. 2009;75: 3093–105. doi:10.1128/AEM.02502-08

4. Duong T, Miller MJ, Barrangou R, Azcarate-Peril MA, Klaenhammer TR. Construction of vectors for inducible and constitutive gene expression in Lactobacillus. Microb Biotechnol. 2011;4: 357–67. doi:10.1111/j.1751-7915.2010.00200.x
