# Supplementary material for: Mucosal Immunogenicity of Genetically Modified Lactobacillus acidophilus Expressing an HIV-1 Epitope within the Surface Layer Protein
Source: PLoS One. 2015 Oct 28;10(10):e0141713. doi: 10.1371/journal.pone.0141713 (PMC4624987; doi:10.1371/journal.pone.0141713)
Supplement: S2 Table — (DOCX) [file pone.0141713.s007.docx]

**S2 Table.** **PCR primers.**

| Primer | Sequence |
| --- | --- |
| AK_54 | AATTGTTAGAATTAGATAAGTGGGCTAGCTTGTGGAACACAAACAGTGATAATCAAACTG |
| AK_55 | CTAGCCCACTTATCTAATTCTAACAATTCTTGCTCGTTACCGGTGAATTTTACATTTGAG |
| AK_56 | GATAAGTGGGCTAGCTTGTG |
| AK_57 | CACAAGCTAGCCCACTTATC |
| AK_62 | GACGAATAAAACTCTAATGAG |
| AK_63 | TTTTAAGCTTCATCTGAGGATAAAGTTGTTTGAT |
| AK_64 | TTTTGGATCCGAATCGAAGTATCAGAAGATCC |
| AK_65 | TGATTTACCACAGTTTGAGTC |
| AK_79 | ATGGACAAACGGTTCAAGCAGTTCCAATTAGACAATTGCACTACAGACTCCGTGATGAAC |
| AK_80 | GTTCATCACGGAGTCTGTAGTGCAATTGTCTAATTGGAACTGCTTGAACCGTTTGTCCAT |
| PCK_1 | TTTTGAATTCAGGAGAAGAATTATGGATAAGAAAGAAGTGAAAAATAG |
| PCK_2 | TTTTCGGCCGTTAGGAAGACACGGATTCCA |
